# Role of Autoimmune Responses in Periodontal Disease

**DOI:** 10.1155/2014/596824

**Published:** 2014-05-25

**Authors:** Soumya Nair, Mohamed Faizuddin, Jayanthi Dharmapalan

**Affiliations:** ^1^Om Dental Clinic, No. 1554, Hebbal 2nd Stage, Mysore 570017, India; ^2^M.R. Ambedkar Dental College and Hospital, No. 1/37, Cline Road, Cooke Town, Bangalore 560005, India

## Abstract

Periodontal diseases are characterized by localized infections and inflammatory conditions that directly affect teeth supporting structures which are the major cause of tooth loss. Several studies have demonstrated the involvement of autoimmune responses in periodontal disease. Evidences of involvement of immunopathology have been reported in periodontal disease. Bacteria in the dental plaque induce antibody formation. Autoreactive T cells, natural killer cells, ANCA, heat shock proteins, autoantibodies, and genetic factors are reported to have an important role in the autoimmune component of periodontal disease. The present review describes the involvement of autoimmune responses in periodontal diseases and also the mechanisms underlying these responses. This review is an attempt to throw light on the etiopathogenesis of periodontal disease highlighting the autoimmunity aspect of the etiopathogenesis involved in the initiation and progression of the disease. However, further clinical trials are required to strengthen the role of autoimmunity as a cause of periodontal disease.

## 1. Introduction

Periodontal diseases are characterized by localized infections and inflammatory conditions where anaerobic Gram-negative bacteria are mainly involved and directly affect teeth-supporting structure. Periodontal disease affects one or more of the periodontal tissues—alveolar bone, periodontal ligament, cementum, and gingiva. The pathogenesis of this disease involves immunological responses leading to tissue destruction and bone loss [1].

Autoimmunity can be defined as breakdown of mechanism responsible for self-tolerance and induction of an immune response against components of the self. Such an immune response may not always be harmful (e.g., anti-idiotype antibodies). However, in numerous (autoimmune) diseases it is well recognized that products of the immune system cause damage to the self (Jiang and Lechler 2003) [2].

In 1965, Brandtzaeg and Kraus were the first to postulate the autoimmune basis in the pathogenesis of periodontal disease. It has been more than 30 years since the concept of autoimmunity has been considered for periodontal disease. An increasing number of reports in the past decade have lent support to the concept of an autoimmune component of periodontal disease [1]. The review is an attempt to focus on the concepts dealing with autoimmunity in periodontal diseases.

## 2. Autoimmunity in Periodontal Disease

### 2.1. Evidence of Autoimmunity in Periodontal Disease: See [3]

There are records of both human as well as animal studies documenting the role of autoimmunity in periodontal disease. The majority of reports deal with the detection of antibodies to host components, in particular, collagen, although antibodies to DNA and aggregated IgG have also been reported (Table 1).

### 2.2. Possible Causes of Autoimmunity [3, 4]

1. Enhanced presentation of self-antigens through increased expression of the molecule associated with antigen presentation, namely, the IgA antigen;
2. altered T helper or T suppressor cell function;
3. polyclonal activation of cells which have the ability, for reasons unclear, to produce autoantibodies;
4. idiosyncrasies of the antigen idiotype network;
5. bacterial or viral cross reactivity with self-antigens leading to production of cross reactive antibodies;
6. genetic predisposition factors.

#### 2.2.1. Increased IgA Expression

In several autoimmune diseases, increase in autoantibody is associated with an increase in molecules which are involved in antigen presentation, in particular on cells which do not normally express these molecules. When these cells which are normally not involved in antigen presentation become involved, it may lead to self-antigen presentation to the immune system and the resultant production of autoantibodies.

#### 2.2.2. Altered T-Helper and T Suppressor Cell Function

In relation to periodontal disease, it has been explained as follows: a reduction in the T cell population of lymphocytes from patients with periodontal disease led to an increase in the B cell component of the spontaneous lymphoblastic proliferation. This would suggest that T suppressor activity would increase in patients with periodontal disease.

#### 2.2.3. Polyclonal Expansion of B Cell Pool: See [4, 5]

Natural autoantibodies (Nab), to a range of self-components, are found in the absence of disease and are presumed to be a natural phenomenon. Such a pool of Nab may be expanded polyclonally by one of the polyclonal B cell activators found in periodontal plaque (LPS) or selectively antigenically by the release of tissue components which expose these natural antigens to the immune system to a greater extent than usual.

#### 2.2.4. Autoantigen Presentation and Autoreactive T Cells: See [3]

All nucleated cells of the body possess class I MHC molecules so that gingival epithelial cell and fibroblast killing reported to occur in periodontal disease in vitro could be mediated by this mechanism of presentation of antigen, in association with class I MHC molecules, to cytotoxic T cells. However, in the lymphocytotoxicity seen in periodontal disease, both allogeneic and syngeneic target cells have been used with similar results. Increased expression of class II MHC molecules has been observed in the surface of basal and suprabasal gingival sulcular epithelial cells in inflamed tissues.

#### 2.2.5. Idiosyncrasies of the Antigen Idiotypic Network [3]

Jerne proposed a network theory of the immune response in which the antigen stimulated the production of antibody exhibiting an idiotype that elicited the formation of an anti-idiotype bearing an “internal image” of that antigen. This latter antibody in turn stimulated the production of anti-anti-idiotype antibody leading to an open ended network.

In periodontal disease there is so far no evidence for existence of anti-idiotype antibodies. The antibodies to immunoglobulin detected in humans and experimental animal periodontal disease are directed to the Fc end of the molecule not the idiotype region.

#### 2.2.6. Viral and Bacterial Infection and the Immune System: See [3, 6]

Viral and bacterial infections may play a role as an exogenous factor triggering an autoimmune response. Activation and clonal expansion of autoreactive lymphocytes is a critical step in the pathogenesis of autoimmune diseases. In experimental models of autoimmunity, disease can be transferred by activated, but not resting, autoreactive T cells [6], indicating that activation of autoreactive T cells is required for the development of autoimmune diseases.The infectious agents act as a stimulating factor such responses. Thus the existence of such a microorganism in periodontal pocket may not only stimulate the production of antibody to this microbe but also stimulate autoantibody production to serum components or degenerated host periodontal tissues.

#### 2.2.7. Genetic Predisposing Factors: See [7–10]

Several investigators have reported an association between a high incidence of selected histocompatibility complex antigens and certain autoimmune diseases, for example, rheumatoid arthritis with HLA-DRw4, systemic lupus erythematosus in association with HLA-DR3 and HLA-B8. This could be an underlying predisposing factor for the development of autoimmune disease. The evidence relating HLA phenotype distribution and the prevalence of periodontal disease has been contradictory. Reports relating HLA phenotype and susceptibility to periodontal disease are mainly associated with HLA class I antigen distribution. A possible role of HLA class I antigens in the development of periodontal disease might be either in relation to cell mediated cytotoxicity and the T cell response or in association with the structural features of the antigens themselves.

Patients with periodontitis juveniles and patient with periodontitis were tissue typed. In the juvenile group, frequencies of tissue type specificities HLA-A9, HLA-A28, and HLABJ'V15 were significantly increased as compared to the findings in the general population. In the periodontitis groups, no significant tissue type deviations were found as discussed by Reinholdt et al. Several theories have been advanced to explain the associations between HLA and a variety of diseases [7].

#### 2.2.8. Role of Antibodies [11]

Perhaps the most pertinent aspect of the antibodies detected in periodontal disease is their role: protective, destructive, or inconsequential. Pathologic changes appear to occur in those circumstances where the otherwise physiologic autoimmune response leads to the initiation of further humoral and or cellular immune activity capable of causing tissue damage or disease. It should be noted that “natural” autoantibody is generally of the IgM class. In adult periodontitis local antibody to collagen type I is predominantly IgG rather than IgM, a reversal of the situation in serum, suggesting that continued antigenic stimulation has led to class switch. In periodontal disease there is alteration in the various subclasses of antibodies, with increasing severity of the disease, such that IgG1 and IgG3 decrease and IgG4 increase. The latter antibody is protective in nature, as it does not form complexes with antigen which activate complement. Studies suggest that the autoantibodies detected in periodontal disease are derived from preexisting natural antibodies and play a physiological role in the elimination of dead cells and damaged tissue constituents that have appeared during the tissue degradation which occurred in periodontal disease. However, the possibility still remains that this system, established to deal with the consequence of tissue damage, may in certain circumstances become excessive and actually contribute to the progress of the disease.

### 2.3. Antineutrophil Cytoplasmic Autoantibodies (ANCA) [12, 13]

ANCAs represent a heterogeneous group of antibodies that target antigens that are primarily present in azurophil granules of polymorphonuclear leukocytes (PMNs). Some ANCA associated diseases are known to coexist during periodontitis in humans. Such diseases include the rheumatoid arthritis and to a lesser extent systemic lupus erythematosus (SLE). More studies are required to explore the link between these conditions and a probable common mechanism in these disease processes.

Exposure of the host to the periodontal pathogens, along with a genetic susceptibility, could trigger ANCA by two different mechanisms. The production of TNF-*α* would sensitize the neutrophils to express its granule contained enzymes, such as MPO and PR-3, which in turn could trigger the production of ANCA. In addition, periodontal pathogens are known to possess a “superantigen” property, where they can directly activate the autoreactive B lymphocytes in a T cell independent and mediated pathway, which can also result in the activation of neutrophils. The activated neutrophils release reactive oxygen radicals, enzymes, and various proinflammatory cytokines, all of which are known to mediate periodontal destruction. ANCA activated neutrophils are also known to delay apoptosis, which can prolong the activity of neutrophils and thereby increase tissue destruction. Delayed apoptosis has been reported in periodontal disease, which can be attributed to ANCA. Furthermore, ANCA is known to have a direct toxic effect on the cells bearing antigens such as endothelial cells, which can result in increased endothelial permeability, a feature common in the inflammatory process.

### 2.4. Role of Natural Killer T Cells in Autoimmunity [14–17]

Human CD1d molecules present glycolipid antigens such as galactosylceramide to CD1d-restricted natural killer T cells. The natural killer T cells appear to associate with CD1d cells, and it was suggested that they have a regulatory role to play in periodontal disease. Autoimmunity has been suggested to be a feature of periodontal disease. Cross reactivity of human heat shock protein (HSP) 60 and* P. gingivalis* GroEL, which is the bacterial homologue has been shown in periodontal disease. HSP 60 specific as well as P.g cross reactive T cells have also been demonstrated to accumulate in periodontitis lesions. The study by Yamazaki et al. suggests that an immune response to autoantigens such as collagen type I or HSP60 may be well controlled by natural killer T cells. A relationship between a deficiency in natural killer cell activity and autoimmune diseases has been cited in mice. An impairment of the subtle balance could be involved in the pathogenesis of periodontal disease. The results, however, did show increase of natural killer T cells in periodontitis, suggesting a functional role for these cells, and because of their ability to secrete rapid amounts of cytokines, they may influence the T helper cytokine response. The role of autoimmunity in chronic inflammation is still not clear. It is possible that autoimmunity is a feature of all chronic inflammation. In this context it has been known for many years that gingival fibroblasts are able to phagocytose collagen such as anticollagen; antibodies may facilitate this phagocytosis and hence removal of broken down collagen. At the same time, an anti-HSP response may enhance the removal of dead and dying cells, such that these autoimmune responses may be a natural part of chronic inflammation. Control of these responses would therefore be essential, hence the increase in regulatory natural killer T cells in periodontal tissues. This concept further illustrates that the role of T cells in periodontal disease may be one of immune homeostasis. Further studies are clearly needed to test this hypothesis and to determine the role of regulatory T cells in periodontal inflammation.

### 2.5. Oral Microbial Heat Shock Proteins [18–20]

When exposed to a wide range of environmental stressors (temperature, pH, redox potential, etc.), prokaryotic and eukaryotic cells respond by inducing or accelerating the synthesis of specific proteins known as stress proteins, including the heat shock proteins (HSPs) which have a high degree of homology. These HSPs are unregulated under stressful conditions. HSPs act as molecular chaperones in the assembly and folding of proteins, and proteases when damaged or toxic proteins have to be degraded. HSPs thus protect cells from damaging effects associated with stressful conditions. The GroELs and, to a lesser extent, the DnaKs of several pathogenic bacteria may become major antigens, since they are strongly expressed under stressful conditions. These HSPs are antigenic, and the recognition of specific epitopes on such highly conserved antigens may contribute to protective immunity or may have pathological autoimmune responses. Because of the similarities between microbial and mammalian HSPs, a humoral response against microbial HSPs may be destructive for the host due to antigenic mimicry leading to autoimmune response.

Three models have been proposed to link microbial infections to subsequent autoimmune reactions involving the HSPs. These models are as follows:

1. molecular mimicry between the microbial HSPs and HSPs or constituent proteins from the host,
2. inflammation induced exposure of cryptic cell epitopes that could be a target for immune reactions,
3. antigen presentation in infected sites leading to chronic immunological reactions.

### 2.6. Role of Autoreactive B Cells in Autoimmunity of Periodontal Disease [3, 4, 11, 21]

B cells upon activation transform into antibody producing plasma cells. There are reasons to anticipate that immunoglobulins produced by plasma cells directed against subgingival plaque may also be autoantibodies directed to host cells. It was observed that the gingival lesions contained a large number of cells that produce either antibodies directed to type I collagen.

In sites of chronic inflammation, polyclonal B cell activators (PBA) are known to exhibit adjuvant activity when combined with foreign antigens. The adjuvant effect of microbial polyclonal B cell activator may be important in anticollagen antibody production, and thus the localization of PBA in periodontal pockets may explain why anticollagen antibody forming cells are restricted to the chronically inflamed periodontal tissues. Interleukin-10 has been reported to selectively promote the expansion of a B lymphocyte lineage which has the propensity for secreting high levels of autoantibody in type I diabetic individuals.

### 2.7. Role of Anti-CCP in Periodontal Disease and RA [22, 23]

Rheumatoid arthritis (RA) is a common, systemic autoimmune disease causing destruction of the joint architecture leading to disability. Etiology of RA remains unknown; accumulating studies have established a strong association between RA and periodontitis (PD). The anti-cyclic citrullinated peptide antibodies (anti-CCP) are produced locally in the inflamed synovium of rheumatoid arthritis (RA) patient. In scientific literature periodontal bacterial DNA in serum and synovial fluid of RA with periodontal disease patients were found. RA and adult periodontitis share common pathogenetic and immunologic mechanisms. One oral pathogen strongly implicated in the pathogenesis of periodontal disease (PD),* Porphyromonas gingivalis*, possesses a unique microbial enzyme, peptidylarginine deiminase (PAD), the human equivalent of which has been identified as a susceptibility factor for RA.* P. gingivalis* is the only oral bacterium that possesses peptidylarginine deiminase activity, which is essential for citrullination of arginine, an RA autoantigen, leading to the formation of immune complexes involved in inflammation and joint destruction seen in RA. The anti-cyclic citrullinated peptide (anti-CCP) autoantibody and citrullinated peptide have been realized to be involved in the breaking of self-tolerance and development of autoimmune in RA. The citrullinated peptide is generated by posttranslational modification (citrullination) of protein-bound arginine by peptidylarginine deiminase (PAD).* Porphyromonas gingivalis* (*P. gingivalis*), the major aetiological agent of PD and the only bacterium known to express a PAD enzyme, has been reported to be significantly associated with RA. It is also confirmed that bacterial PAD produced by* P. gingivalis* has the capacity of deiminating arginine in fibrin found in the periodontal lesion. What is more, it has been demonstrated that citrullination of HLA binding peptide causes a 100-fold increase in peptide-MHC affinity and leads to the activation of CD4(+) T cells in HLA DRB1 0401 transgenic mice. Therefore, we postulate that* P. gingivalis* may play a crucial role in the pathogenesis of periodontitis-associated RA.* P. gingivalis*, which colonizes in the oral cavity, produces PAD enzyme continuously that leads to the citrullination of RA autoantigen such as fibrin in synovium joint. These PAD engendered antigens, presented in association with major histocompatibility complex (MHC) molecules by antigen-presenting cells (APC), ultimately lead to production of the anti-CCP antibody. The anti-CCP antibodies form immune complexes with citrullinated proteins, which can be bound by inflammatory cells via their Fc receptors. The roles of these immune complexes and inflammatory cells are mediated by a complex cascade involving complement activation. These mechanisms result in a release of mediators of inflammation and joint destruction ultimately leading to the onset of RA. This hypothesis reveals that oral bacterial infection may play a role in peptide citrullination which might be involved in loss of self-tolerance and development of autoimmunity in RA.

## 3. Autoimmunity as a Component in Various Periodontal Diseases

### 3.1. Host Immune Response Linked to the High Risk of Periodontal Disease in Diabetics [24–26]

Diabetic patients are at a higher risk of developing periodontitis is a known fact, however the mechanism is not fully understood. An autoimmune component cannot be fully ruled out as per the Mahamed et al. study. This research study shows that the autoimmune environment and CD4+ T cells display an unusual hyperactive response when mounting an antibacterial immunity to oral microbial assaults in the experimental diabetic NOD (non obese diabetic) mice, which is similar to human type 1 diabetes. These findings will lead to a new understanding of the potential causes of the high rates of microbial infections in diabetics and future treatments for both periodontal/dental care and medical risk factor management. This study clearly describes the impact of the autoimmunity environment to anaerobic infection in an experimental periodontitis model of type1 diabetes. Moreover, these cells in high-risk diabetic patients may open a new door for the therapeutic potential of treating periodontal disease. In another study a cytokine, interleukin (IL)-10, has been reported to selectively promote the expansion of a B lymphocyte lineage (CD5/LY1/B1) which has the propensity for secreting high leves of autoantibody in diabectic patients.

### 3.2. Role of Autoimmunity in Aggressive Periodontitis [26]

Aggressive forms of periodontitis have been intensively investigated due to their aggressiveness, quick progression, and occurrence in adolescents and young individuals. Periodontopathogenic bacteria have been implicated in the pathogenic ability of AgP, and there is evidence in literature that bacterial toxins and autoimmune mechanisms are likely involved in the pathogenesis of AgP. In a recent study carried out, the presences of autoantibodies directed against ECM components were found in the sera of patients. However it does not necessarily mean that these autoantibodies primarily participate in the destructive mechanism of periodontal tissue. Their presence could be a coadjuvant factor rather than the cause of these lesions. It was demonstrated that self-antigens may be altered in the course of infection. Furthermore microbial antigens exhibit molecular mimicry with host self-antigens and thus facilitate the production of autoantibodies.

### 3.3. Desquamative Gingivitis [1]

It is not a specific disease entity, but a gingival response associated with a variety of conditions. A variety of conditions affect the gingiva, either in part or totally. The gingiva consists of the epithelium, the connective tissue, and the basement membrane separating the two. Consequently, various conditions can involve each component specifically or totally, or, in some cases, can involve specific aspects of each component, such as certain layers of epithelium. In many of these conditions some type of inflammatory process is involved, either acute or chronic. Autoimmune mechanisms appear to be a prominent aspect of etiology in many patients. Thus, similar to periodontal disease, inflammation and the immune response are involved in pathogenesis of the mucocutaneous lesions of the gingiva.

## 4. Discussion

Periodontal disease is characterized by the loss of the normal supporting tissues of the teeth and a humoral and cellular immune response to bacterial antigens of dental plaque which accumulates at the dentogingival junction. However, till date with so much advances made in the field of periodontics, the etiopathogenesis of this infectious disease is still an enigma. It has been more than 30 years since the concept of autoimmune pathogenesis for periodontal disease was considered. An increasing number of reports in the past decades has lent support to the concept of an autoimmune component of periodontal disease. Three aspects of the phenomenon warrant consideration: firstly, the evidence which has accumulated over the past in relation to autoimmunity; secondly, the mechanism by which such autoimmunity may arise; and lastly, the possible role of such an abnormal immune response in the natural history of the disease.

Perhaps the most pertinent aspect of the antibodies detected in periodontal disease is their role: protective role, destructive, or inconsequential part in the pathogenesis of the disease. Just the mere presence of antibodies to self-components does not necessarily lead to disease. Pathologic changes appear to occur in those circumstances where the otherwise physiological autoimmune response leads to initiation of further humoral and/or cellular immune activity capable of causing damage or disease. To some extent the pathologic potential of the autoantibodies can be determined by knowledge of their nature, for example, their class and possibly subclass. It should be noted that natural autoantibodies are generally IgM class. In adult periodontitis there is local antibody to collagen type I, which is predominantly IgG type rather than IgM, a reversal of what is there in serum, suggesting that continued antigenic stimulation could lead to a class switch and further an increase in IgG4 and decrease in IgG1 and IgG3 occurs. IgG4 is protective in nature in that it does not form complexes with antigen to stimulate the complement. These findings suggest that the autoantibodies detected in periodontal disease are derived from preexisting natural antibodies and play a physiological role in elimination of dead cells and tissues that have occurred during tissue degradation. However, the possibility still remains that this system, established to deal with the consequences of tissue damage, may in certain circumstances become aggressive to actually contribute to the disease process [1, 3, 11].

Considering the role of antinuclear cytoplasmic antibodies (ANCA) in periodontal disease, it is seen that exposure of the host to the periodontal pathogens, along with a genetic susceptibility, could trigger ANCA. The production of TNF-a would sensitize or prime the neutrophils which in turn could trigger the production of ANCA. In addition, periodontal pathogens are known to possess a “superantigen” property, which can also result in the activation of neutrophils. The activated neutrophils release reactive oxygen radicals, enzymes, and various proinflammatory cytokines, all of which are known to mediate periodontal destruction. ANCA activated neutrophils are also known to delay apoptosis, which can prolong the activity of neutrophils and thereby increase tissue destruction. Delayed apoptosis has been reported in periodontal disease. Furthermore, ANCA is known to have a direct toxic effect on the cells bearing antigens such as endothelial cells, which can result in increased endothelial permeability, a feature common in the inflammatory process. All said and done, to evaluate the exact role of ANCA in periodontal disease pathogenesis, further human clinical trials are required [12, 13].

The HSPs are antigenic, and the recognition of specific epitopes on such highly conserved antigens may contribute to protective immunity or may have pathological autoimmune responses. Because of the similarities between microbial and mammalian HSPs, a humoral response against microbial HSPs may be destructive for the host due to antigenic mimicry leading to autoimmune response.

Studies show that an immune response to autoantigens such as collagen type I or HSP60 may be well controlled by natural killer T cells. However downregulation of CD1d in murine T cells by P.g has been demonstrated implying that even though there may be higher numbers of natural killer T cells in periodontitis, they may not be functional. Furthermore the exact role of these natural killer cells in autoimmunity is still not clear [15].

Finally we come to the genetic predisposition towards autoimmunity in terms of HLA [24, 26].

The HLA system probably contains so-called immune response (Ir) genes which control the specific responsiveness toward a variety of antigens. These genes are most likely in linkage disequilibrium with the HLAA and B genes, and, in one instance, it is possible that an Ir gene controlling autoimmunity could explain the findings; in the other instance, absence of a specific Ir gene could lead to chronic infection. Some microbial antigens may resemble some HLA antigens which would make individuals carrying these antigens less capable of reacting immunologically against the microorganisms in question. Finally, it seems as if HLA antigens serve a biological function as receptors for foreign antigens which they present in an adequate way for immunocompetent cells. This mechanism could lead both to autoimmunity and, if the relevant HLA antigen was absent, susceptibility to infection. It is impossible to decide which mechanism operates in the possible association between HLA and juvenile periodontitis.

Just as so many aspects of the etiopathogenesis of periodontal disease remain unsolved, this particular aspect of role of autoimmunity in periodontics is still quite intriguing. Lots of pieces of this jigsaw puzzle still do not fit in accurately. However, our attempt to understand the natural history of this disease process still has a long way to go. Probably once this is properly understood, the etiopathogenesis may become more coherent and help us enhance our cognizance of this chronic inflammatory condition. Understanding the exact role of autoimmunity can have serious therapeutic implications in the treatment of periodontitis either in the form of development of a vaccine or some miracle drug [27–30].

## 5. Conclusion

Periodontal disease being a chronic inflammatory disease has a complex etiopathogenesis which still remains not completely understood. The present review has thrown light on the autoimmune component of the disease, which may be somewhere underplayed or ignored in its role in the etiopathogenesis of this disease. The objective of the review is to highlight this particular aspect of the disease so that further research can be conducted to get a more clear picture of the autoimmune component of this disease and how we can apply it in therapeutics. There is more than enough evidence to quote an autoimmunity aspect to periodontal disease, which could be the main reason why the initiation and progression of this disease is still unclear.

## Figures and Tables

**Table 1:** 

Humoral	Cellular
(1) Antibody to aggregated IgG (rheumatoid factor) detected in gingiva and saliva	(1) Lymphocytotoxicity shown for oral epithelial cells
(2) Antibody levels to double stranded DNA were elevated in dog gingival	(2) Histological damage observed in gingival human fibroblasts with associated mononuclear cells
(3) Elevated levels of serum antibody to human collagen type I	(3) Lymphocytotoxic killing of autologous fibroblasts with associated mononuclear cells
(4) IgM, IgA rheumatoid factor antibody to human collagen types I and III produced by mononuclear cells	(4) Lymphoblastogenesis detected to native and denatured collagen
(5) Serum IgG antibodies to native and denatured types I and III bovine collagen detected in juvenile periodontitis	(5) Cellular immunity to proteoglycan detected in periodontal disease patients
